# GoodReports: developing a website to help health researchers find and use reporting guidelines

**DOI:** 10.1186/s12874-021-01402-x

**Published:** 2021-10-17

**Authors:** Caroline Struthers, James Harwood, Jennifer Anne de Beyer, Paula Dhiman, Patricia Logullo, Michael Schlüssel

**Affiliations:** 1 UK EQUATOR Centre, Centre for Statistics in Medicine, NDORMS, University of Oxford, Oxford, UK; 2 NIHR Oxford Biomedical Research Centre, Oxford University Hospitals NHS Foundation Trust, Oxford, UK

**Keywords:** Standards, Software, Reporting guidelines, Education, Reproducibility

## Abstract

**Background:**

Th EQUATOR Network improves the quality and transparency in health research, primarily by promoting awareness and use of reporting guidelines. In 2018, the UK EQUATOR Centre launched GoodReports.org, a website that helps authors find and use reporting guidelines. This paper describes the tool’s development so far. We describe user experience and behaviour of using GoodReports.org both inside and outside a journal manuscript submission process. We intend to use our findings to inform future development and testing of the tool.

**Methods:**

We conducted a survey to collect data on user experience of the GoodReports website. We cross-checked a random sample of 100 manuscripts submitted to a partner journal to describe the level of agreement between the tool’s checklist recommendation and what we would have recommended. We compared the proportion of authors submitting a completed reporting checklist alongside their manuscripts between groups exposed or not exposed to the GoodReports tool. We also conducted a study comparing completeness of reporting of manuscript text before an author received a reporting guideline recommendation from GoodReports.org with the completeness of the text subsequently submitted to a partner journal.

**Results:**

Seventy percent (423/599) of survey respondents rated GoodReports 8 or more out of 10 for usefulness, and 74% (198/267) said they had made changes to their manuscript after using the website. We agreed with the GoodReports reporting guideline recommendation in 84% (72/86) of cases. Of authors who completed the guideline finder questionnaire, 14% (10/69) failed to submit a completed checklist compared to 30% (41/136) who did not use the tool. Of the 69 authors who received a GoodReports reporting guideline recommendation, 20 manuscript pairs could be reviewed before and after use of GoodReports. Five included more information in their methods section after exposure to GoodReports. On average, authors reported 57% of necessary reporting items before completing a checklist on GoodReports.org and 60% after.

**Conclusion:**

The data suggest that reporting guidance is needed early in the writing process, not at submission stage. We are developing GoodReports by adding more reporting guidelines and by creating editable article templates. We will test whether GoodReports users write more complete study reports in a randomised trial targeting researchers starting to write health research articles.

**Supplementary Information:**

The online version contains supplementary material available at 10.1186/s12874-021-01402-x.

## Background

Around 80% of articles reporting health-related research do not include enough detail for a reader to fully understand, assess, and replicate the methods and results [1]. Reporting guidelines aim to solve this problem by specifying the minimum information authors need to include when writing up their research for publication. Reporting guideline documents often include a checklist, which many medical journals ask authors to submit alongside their manuscripts as evidence they have included all the necessary information.

The EQUATOR (Enhancing Quality and Transparency of Health Research) Network is an international initiative that has been providing support for the dissemination and use of reporting guidelines since 2006. The UK EQUATOR Centre makes reporting guidelines more accessible by maintaining a centralised, searchable database alongside resources and training to support their use [2]. Despite the work of EQUATOR and many other organisations, such as NC3Rs, Cochrane, ICMJE, WAME, EASE, and COPE [3–8], to promote the use of reporting guidelines, reporting quality remains poor [9] and use of even the most popular guidelines remains low [10, 11].

A scoping review of interventions to improve adherence to reporting guidelines found a lack of practical training on how to use them and that guidelines were not easy to access or understand [12]. There are over 400 reporting guidelines in the EQUATOR database, which differ in the amounts of instruction they provide. Authors may struggle to choose an appropriate guideline. Many are published behind paywalls and in unusable formats, such as PDF checklists that cannot be filled in.

To address these issues, the UK EQUATOR Centre has created GoodReports.org [13], a website that helps authors select the most appropriate reporting guideline for their study and gives them immediate access to a user-friendly checklist. Authors can fill in the checklist online and download it to include with their journal submission.

This paper describes the development of GoodReports.org, and observations of user attitudes, experience, and behaviour. The UK EQUATOR Centre has partnered with Penelope.ai [14], a software company already providing a manuscript-checking service to *BMJ Open*. This partnership allowed us to quickly drive traffic to the GoodReports website and observe whether authors who completed a GoodReports checklist and submitted it to the journal with their article added information to their manuscripts. We also gathered qualitative user feedback from authors using Penelope to assess whether this workflow to access reporting checklists is feasible and how future development of GoodReports could better serve author needs.

## Methods

### Developing GoodReports.org

The GoodReports website has two main features: authors can complete a questionnaire about their study to receive a reporting guideline suggestion, then can immediately access reporting checklists to fill out on- or offline. Each checklist includes clear instructions, and each reporting item is linked to an explanation of why that item is needed and examples of good reporting, whenever guideline developers have provided such information.

We decided to include reporting guidelines that cover the main generic study designs and are commonly recommended by journals. We started with the 13 popular reporting guidelines highlighted on the EQUATOR homepage. Although published as one reporting guideline, STROBE covers three observational study designs and is made available by the STROBE development group as three separate checklists. We included all three. We added STREGA for genetic association studies [15] as it is included in the *BMJ Open* guide for authors. The 16 reporting guidelines included in GoodReports are shown in Table 1.

**Table 1:** List of reporting guidelines in the GoodReports database [15–28]

	Name	Study type
1	ARRIVE	Bioscience using laboratory animals^a^
2	CARE	Case reports and data from the point of care.
3	CHEERS	Economic evaluations of health interventions
4	CONSORT	Parallel group randomised trials^a^
5	MOOSE	Meta-analysis of observational studies in epidemiology
6	PRISMA	Systematic reviews and meta-analyses^a^
7	PRISMA-P	Systematic review and meta-analysis protocols
8	SPIRIT	Protocol items for clinical trials^a^
9	SQUIRE	Quality improvement in health care
10	SRQR	Qualitative research
11	STARD	Diagnostic accuracy^a^
12	STREGA	Genetic association studies
13	STROBE case control	Case-control studies^a^
14	STROBE cohort	Cohort studies^a^
15	STROBE cross sectional	Cross-sectional studies^a^
16	TRIPOD	Developing, validating, or updating a prediction model

^a^ GoodReports entry includes link to explanation and examples

**Fig. 1:**
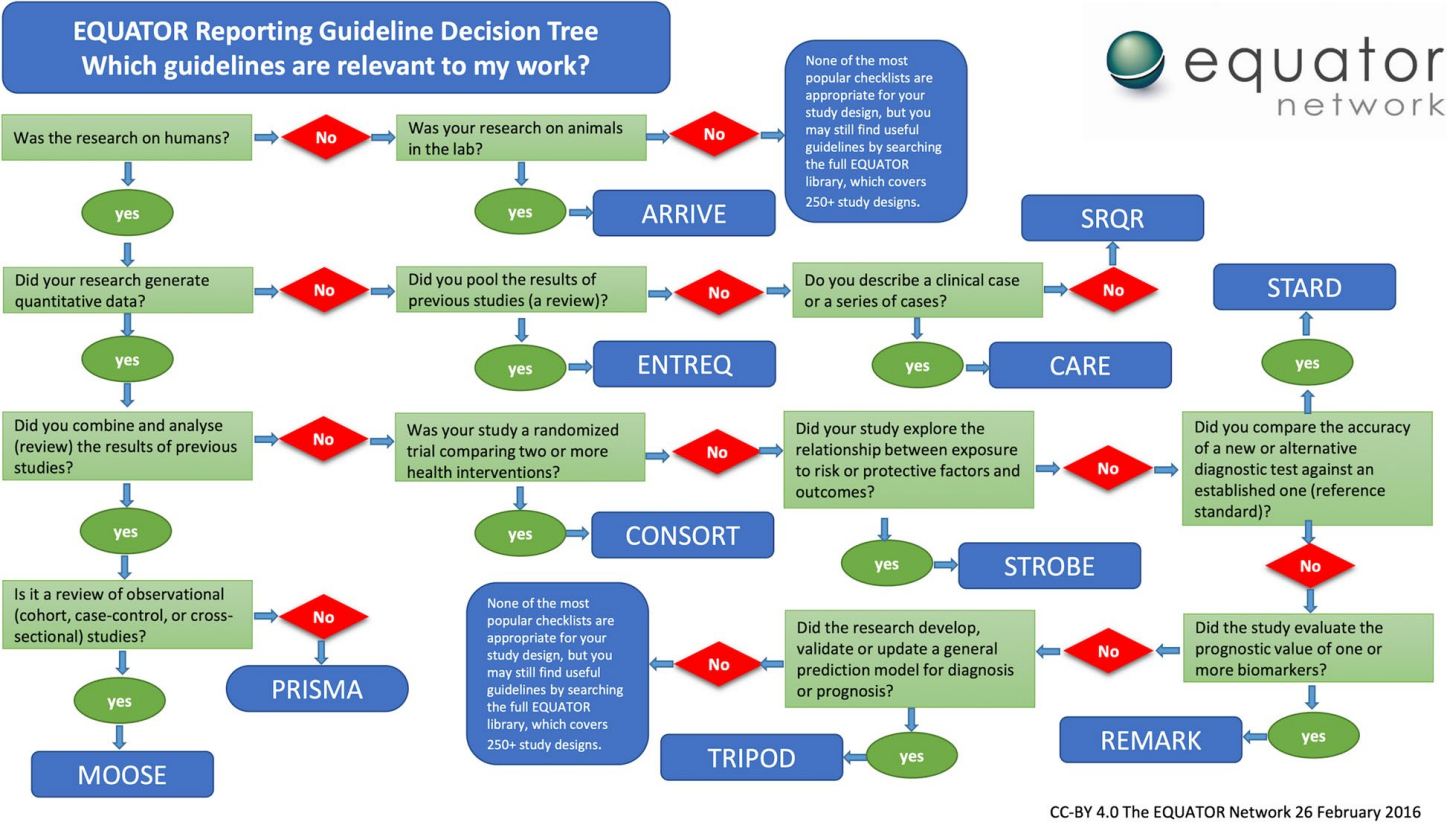
EQUATOR reporting guideline decision tree (“wizard”) developed 2016. Available from the EQUATOR website [29]

We designed a questionnaire to help authors find which, if any, of these 16 reporting guidelines applied to their work. The questionnaire was based on a previous “wizard” developed in 2016 that used a simple yes/no decision tree structure (Fig. 1).

**Fig. 2:**
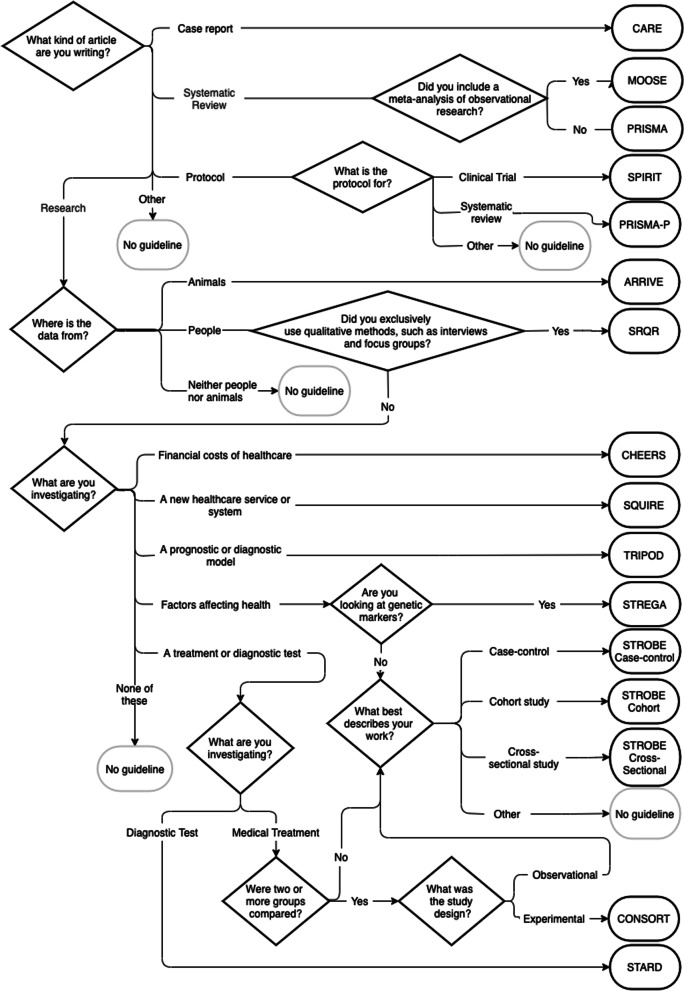
The GoodReports checklist finder questionnaire

We adapted the original questionnaire to our new set of guidelines (Fig. 2).

We reduced the use of research and methods jargon as much as possible to improve accessibility and clarified some of the questions in response to initial user feedback. We used multiple-choice options to keep the decision tree short and easy to navigate. The publishers’ copyright licence for MOOSE [20] and SRQR [25] did not automatically allow us to reuse the content to create an openly accessible online checklist. We were granted permission to do so on payment of a licence fee for one and two years respectively, which covered the duration of this study.

### Reaching users

GoodReports.org went live in January 2018. We used Penelope.ai [14], a company owned by co-author JH, to attract users. Penelope.ai provides software to journals that automatically checks new submissions and gives immediate feedback to authors to help them meet journal requirements. Penelope.ai allowed us to collaborate with *BMJ Open*, one of Penelope.ai’s customers, to capture authors in the process of submitting their articles for publication. All authors submitting to *BMJ Open* can opt to use Penelope.ai, but it is not mandatory.

From January 2018, the Penelope.ai upload form was amended to include the GoodReports guideline recommendation questionnaire (Fig. 2). Authors who used Penelope.ai therefore had to answer the checklist finder questionnaire before uploading their manuscript. When appropriate, Penelope.ai’s feedback report on their manuscript included a recommendation to use a reporting guideline and a link to the associated checklist on GoodReports.org.

Below is a representative author journey from one of Penelope.ai’s client journals, *BMJ Open*.

1. Authors begin their submission on BMJ Open:
  https://bmjopen.bmj.com/pages/authors/
2. Authors receive an option for an automated manuscript check:
  https://app.penelope.ai/manuscript-check/q/bmjopen
3. Authors that opt for an automated check answer a few questions about their work and view their feedback online:
  https://app.penelope.ai/submissions/demo/
4. Depending on the information authors give when uploading to Penelope.ai, their feedback may include an instruction to complete a reporting guideline on
  https://www.goodreports.org

#### Individual user feedback

From 25 January 2018 to 6 November 2019, all Penelope.ai users who received a reporting checklist recommendation as part of their manuscript report were sent an automated email survey a day later about their experience of being recommended to use a reporting guideline and using the GoodReports website. We used Typeform.com [30] to collect survey responses. With the first question embedded in the email, we asked users:

1. “You were recently advised to complete a checklist at www.goodreports.org. How useful did you find the checklist?” (rating scale: 0 (least useful) to 10)
  1. If a rating of 7 or lower: “Can you explain why you gave a rating of [number]?” (multiple choice. We set 7 as the cut-off for this question as we anticipated that our median rating would be 8/10)
    The checklist items were not relevant to my work
    The checklist was too long
    The checklist was confusing
    The website was confusing
    Other (free text)
2. “How could we make www.goodreports.org more useful?” (free text)
3. From December 2018: “After using the checklist did you make any changes to your manuscript?” (yes/no)
  1. If yes, “What did you change?” (free text)
  2. If no, “Why didn’t you make any changes?” (free text)

Quantitative responses are reported as counts. The free-text responses for question 3.1 and 3.2 were coded by JH using Excel, allowing general themes to emerge which were then discussed by CS and JH. We report the themes, their frequency, and representative quotes.

#### Performance of the questionnaire in helping authors find the most appropriate reporting guideline for their work

To determine whether the checklist finder questionnaire generally led users to an appropriate checklist, we randomly selected 100 manuscripts from all of those uploaded to Penelope.ai by *BMJ Open* authors for an automatic manuscript check between 25 January 2018 and 16 February 2019.

CS and JH separately read the titles, abstracts, and methods section of each manuscript and decided which, if any, of the 16 guidelines in the GoodReports database should have been recommended. They compared recommendations and resolved conflicts through discussion. They were blinded to the GoodReports checklist finder recommendation up to this point. The consensus assessor recommendation was then compared with the recommendation that authors received from the checklist finder. Where the recommendations differed, the assessors examined the questionnaire responses and the corresponding manuscripts together and considered the most likely reason for the discrepancy.

We report the percentage of manuscripts where the checklist finder questionnaire and assessor recommendations matched and possible reasons for mismatches. No statistical tests were done, as the purpose was to identify how the questionnaire could be improved before a more rigorous evaluation.

### Use of GoodReports, checklist submission rates, and manuscript completeness

*BMJ Open* is an existing customer of Penelope.ai. The Editor-in-Chief agreed to allow us access to submitted manuscripts to gather initial data on author behaviour when receiving a GoodReports reporting checklist recommendation as part of the Penelope.ai manuscript check.

We were interested in whether authors exposed to GoodReports included completed reporting checklists with their submission, and whether the exposure led authors to add missing information to their manuscripts before submission.

We collected data from all newly submitted manuscripts checked by *BMJ Open* staff on 9, 10, 11, 25, 28, and 29 May 2018. These dates were selected by the journal and determined the sample size. It was not practical for the journal to increase the length of the data collection period. We only included manuscripts checked for the first time on these dates. We excluded manuscripts that had been first submitted before those dates, returned to the author for corrections, and resubmitted.

#### Exposure to GoodReports.org and rates of submission of a completed reporting checklist

*BMJ Open* shared some of the data they collect routinely as part of the editorial process. This data included whether the submission had previously been checked, and notes from the technical editor about unmet journal requirements. Data on whether the author had included a reporting checklist in their submission was determined by the journal policy on enforcement of checklists and the editor’s notes. We were therefore able to count the number of “checklists noted to be missing.”

We split submissions into two groups: those whose authors had opted to check their manuscript with Penelope.ai before submission and received a checklist recommendation, when appropriate, and those whose authors had opted not to use the checker. JH identified whether an author had used Penelope.ai by searching the Penelope.ai logs for the author’s email address and cross-referencing the manuscript’s file names and titles, without knowing whether a reporting guideline had been submitted for that manuscript.

We report the proportion of manuscripts where a checklist had been flagged as missing for each group.

#### Completeness of reporting before and after using a GoodReports reporting checklist

We reviewed whether authors that used and submitted a reporting guideline checklist from GoodReports.org had changed their manuscript and improved the completeness of their reporting as a result.

We started with the subset of manuscripts from the study on submission rates that had:

1. been checked by Penelope.ai before submission to *BMJ Open,*
2. not withdrawn their submission from *BMJ Open*, and
3. included a reporting guideline checklist from GoodReports.org.

We conducted a descriptive before-and-after study on the included manuscripts. Completeness of reporting was described in the version that was uploaded to Penelope.ai for an automatic pre-submission check (the “before” version) and in the version subsequently submitted to *BMJ Open* (the “after” version).

Assessors checked whether the “after” version submitted to BMJ Open contained the same information as the “before” version or whether the author had added information.

We excluded manuscripts submitted with checklists obtained elsewhere, such as the EQUATOR Network website or the journal website, to reduce the chance that the authors of manuscripts in our “before” group had already used a checklist before visiting GoodReports.org.

JH redacted the title and methods sections of the “before” and “after” versions so that no personal information was shared with assessors. The “before” versions were all in .docx format, so text could be copied and pasted into a fresh Microsoft Word file. The “after” versions were PDFs as *BMJ Open* automatically converts submissions into PDF and adds watermarks, line numbers, and footers. JH split PDF files into smaller files containing only the title and methods sections for data extraction. These differing file formats meant that assessors were not blinded to whether the manuscript was the “before” or “after” version.

Five assessors (JdB, MS, PD, PL, and AK) were allocated a selection of manuscript pairs and assessed the methods sections of the “before” and “after” versions. Each manuscript pair was assessed by three data extractors. CS assessed the titles of all 20 manuscripts.

The assessors checked whether the “before” version submitted to Penelope.ai contained adequate information for each item in the methods section of the appropriate reporting checklist. Each item was assessed as present, absent, unclear/partial, or not applicable to that manuscript.

The assessors then checked the “after” version for any added information. Each item was assessed as “no change” or “added information”. As each reporting guideline has a different number of items, we report the counts as percentages.

### Ethics and consent

In accordance with the University of Oxford’s policy on the ethical conduct of research involving human participants and personal data [31], ethical approval and informed consent were not required. We used data collected as part of our partner PNLP Ltd.’s optional manuscript checking service, and during the normal course of *BMJ Open*’s editorial procedures. In accordance with the personal data protection policies of our partners, all data was anonymised before it was shared with the research team.

## Results

### Individual user feedback

Between 16 January 2018 and 6 November 2019, 16,812 people received feedback from Penelope.ai, 10,729 of whom were recommended a reporting checklist on GoodReports.org as part of their feedback. Nearly 40% (4,182/10,729) of these users clicked the link to visit the GoodReports.org. All 10,729 users were sent an email survey one day after using Penelope.ai asking about their experience of using GoodReports.org. We received 623 responses.

#### Usefulness ratings

**Fig. 3:**
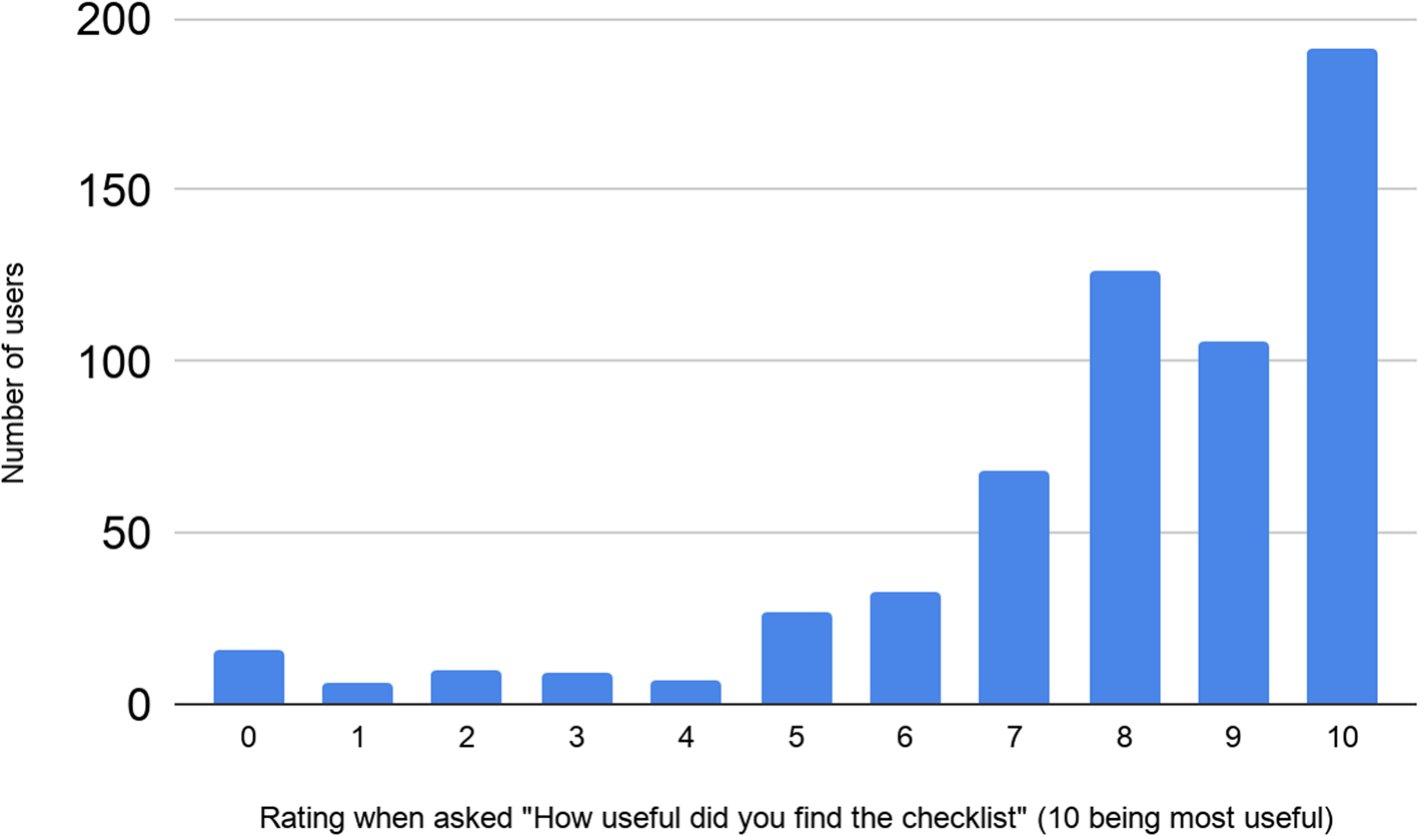
Responses to “How useful did you find the checklist” (10 = most useful)

Most of the responders (599/623) answered the question “How useful did you find the checklist?” Fig. 3 shows the distribution of ratings.

Only 176/599 of responders who answered question 1 (30%) rated GoodReports.org 7 or below for usefulness. Table 2 shows the responses of the 159/176 responders who explained why they gave this lower rating using our multiple-choice options and free text.

**Table 2:** Users’ reasons for rating www.goodreports.org 7/10 or lower. Users could select more than one multiple-choice option

Why did you rate www.goodreports.org a [e.g., 6]?	
The checklist was too long	62/159 (39%)
The checklist was confusing	51/159 (32%)
The checklist items were not relevant to my work	50/159 (31%)
The website was confusing	6/159 (4%)
Other Free text answers *• Too difficult to complete the list points* *• We have already done the Prisma checklist. These two checklists overlap each other.* *• No clue what you're talking about. Nobody asked me to do a checklist.* *• Some of the checklist items were confusing* *• Some items are not relevant* *• Already addressed in my paper.* *• Checklist assume too much about the nature of ‘good’ work* *• Check list is the same as the one on the journal guidelines* *• I completed the whole form and then when I clicked the button at the end it deleted all my answers* *• Some of the checklist were not relevant to my work and i taught it would have looked at my discussion in detail* *• The checklist is still rather broad* *• The checklist mentioned several items which were included in the article (e.g. corresponding author, subheadings)* *• Checklist mentioned that items were missing when they were present but with a slightly different spelling e.g. Conflicts of Interest instead of Conflicts of Interests* *• Not listed in the journal’s Instructions to authors* *• It was incorrect* *• Good* *• Some items were not available to my article.* *• 7 is a decent rating* *• Fair, the checklist differs with different countries* *• COREQ checklist was used.*	20/159 (13%)

#### Suggestions for improvement

274/623 respondents (44%) responded to the question “How could we make www.goodreports.org more useful?” Of these, 71 (26%) gave a neutral response with no suggestion (e.g., “Not sure”), 57 (21%) were general compliments (e.g., “Easy to navigate and very useful”), 50 (18%) were comments about the workflow through which they had encountered GoodReports.org (e.g., from *BMJ Open* or via the Penelope.ai manuscript checker), and 6 (2%) were criticisms of reporting checklists in general (e.g., “Blind checklists are not relevant to most work”).

The remaining 90/274 (33%) responses included 93 actionable suggestions for improvement, with some responses including more than one suggestion. See Table S1 in the supplementary file which shows the broad themes in these suggestions and representative comments.

#### User feedback on completeness

Between 4 December 2018 and 6 November 2019, we received 267 responses to the question “After using the checklist did you make any changes to your manuscript?” Most respondents (198/267, 74%) said they had made changes, and 69 (26%) said they had not. See Table S2 in the supplementary file for themes and comments about changes made or reasons for not making changes.

### Performance of the questionnaire in helping authors find the most appropriate reporting guideline for their work

Between 25 January 2018 and 16 February 2019, 5,831 authors submitting to *BMJ Open* elected to check their work with Penelope.ai before completing submission. We randomly selected 100 of these manuscripts and compared our recommendations of reporting guidelines with that recommended by the GoodReports checklist finder.

Seventy-three (73/100) of the questionnaire recommendations were considered correct: 57/100 manuscripts were recommended an appropriate guideline and 16/100 were correctly told that no appropriate guideline existed.

Twenty-seven of the questionnaire recommendations were considered incorrect: 21 cases where we thought the wrong checklist had been recommended and 6 cases where we thought there was an appropriate checklist, but none was recommended

In the 27 cases where we assessed that the wrong recommendation had been made, we compared the authors’ responses to the questionnaire with the associated manuscript for clues as to how the questionnaire could be improved. See Table S3 in the supplementary file for a summary of possible reasons for inappropriate recommendations, and potential solutions

### Exposure to GoodReports.org and rates of submission of a completed reporting checklist

**Fig. 4:**
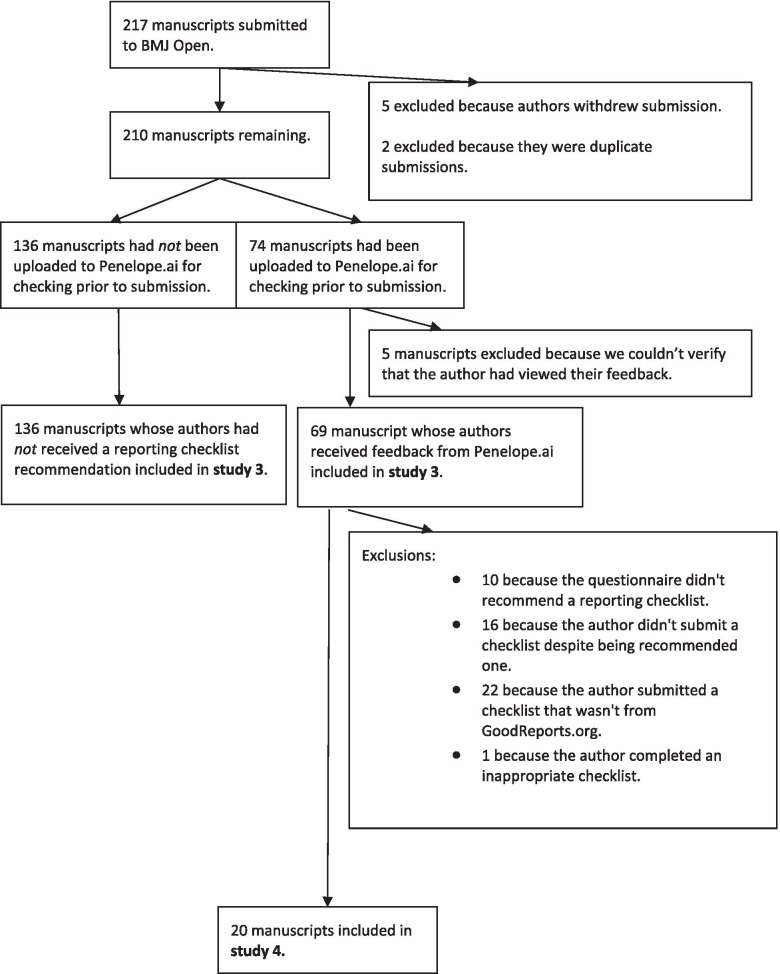
Flow diagram showing how manuscripts were selected for rates of checklist submission and for the before-and-after completeness study

Figure 4 shows how we selected manuscripts to review rates of checklist submission when GoodReports is used, and to review completeness of reporting. Over the six data collection days in May 2018, 217 newly submitted manuscripts were checked by the *BMJ Open* editorial team. Five manuscripts were excluded because the author withdrew their submission between when the manuscript was checked and when *BMJ Open* exported the data to share with us. As *BMJ Open* deletes submission data at withdrawal, we could not check whether these manuscripts had used Penelope.ai. We also excluded two manuscripts that were flagged as duplicate submissions that had already been checked once before by *BMJ Open* staff.

We matched 74 of the remaining 210 submissions (35%) to Penelope.ai uploads. We excluded manuscripts from 5 authors who did not view their feedback (which may have included a reporting guideline recommendation) on Penelope.ai. We therefore analysed compliance with checklist submission using 205 manuscripts, 69 that were exposed to GoodReports via Penelope.ai and 136 that were not.

The editorial office checked each submission to determine whether a checklist was required and, if so, whether it had been submitted. Of the authors that did not use Penelope.ai, *BMJ Open*’s editorial team had to chase 41/136 (30%) of them for a checklist. Of the authors that did use Penelope.ai, the editorial team had to chase 10/69 (14%).

### Completeness of reporting before and after using a GoodReports reporting checklist

Of the 69 manuscripts that had used Penelope.ai, 10 were excluded because the GoodReports questionnaire did not make a checklist recommendation, 16 because they did not submit a checklist to the journal, 22 because the checklist submitted alongside the manuscript had not come from GoodReports.org, and 1 because it was submitted with the wrong GoodReports checklist. We therefore assessed 20 manuscripts (Fig. 4).

Table 3 compares the completeness of reporting in the manuscript version submitted to Penelope.ai before completing a GoodReports reporting checklist with the version submitted to *BMJ Open* after completing the recommended checklist.

**Table 3:** Completeness of reporting in manuscripts before and after completing a GoodReports checklist

	Applicable reporting checklist	Number of method items in checklist	Items reported *before* completing a reporting checklist	Items reported (or reported more fully) *after* completing a reporting checklist	Number of reporting items improved
1	PRISMA-P	15	12	12	0
2	PRISMA-P	15	12	14	2
3	PRISMA-P	15	13	13	0
4	SRQR	11	6	7	1
5	SPIRIT	25	18	18	0
6	SPIRIT	25	12	12	0
7	SPIRIT	25	14	20	6
8	SPIRIT	25	15	16	1
9	CONSORT	16	10	11	1
10	STROBE Cohort	14	4	4	0
11	STROBE Cohort	14	10	10	0
12	STROBE Cohort	14	10	10	0
13	STROBE Cross-sectional	13	10	10	0
14	STROBE Cross-sectional	13	5	5	0
15	STROBE Cross-sectional	13	6	6	0
16	TRIPOD	18	11	11	0
17	STARD	17	8	8	0
18	MOOSE	8	6	6	0
19	MOOSE	8	2	2	0
20	SQUIRE	8	0	0	0

*CONSORT* Consolidated Standards of Reporting Trials, *MOOSE* Meta-analyses Of Observational Studies in Epidemiology, *PRISMA-P* Preferred Reporting Items for Systematic reviews and Meta-Analyses for Protocols, *SRQR* Standards for Reporting Qualitative Research, *SPIRIT* Standard Protocol Items: Recommendations for Interventional Trials, *STARD* Standards for Reporting Diagnostic Accuracy, *STROBE* STrengthening the Reporting of OBservational studies in Epidemiology (sub-checklists for cohort, cross-sectional, and case-control studies), *TRIPOD* Transparent Reporting of a multivariable prediction model for Individual Prognosis Or Diagnosis

Five of the 20 (25%) manuscripts improved their methodological reporting after having completed a reporting checklist on GoodReports.org. Of these, 3 added information for 1 reporting item, 1 added information for 2 items, and 1 added information for 6 items. Three of the 5 (60%) improved manuscripts were protocols, whereas 7/20 (35%) of the full sample were protocols.

No manuscript completely described all methodological reporting items from the recommended reporting checklist, either before or after completing a checklist. One manuscript failed to address any items from its checklist. On average, manuscripts described 57% (SD 22%) of necessary reporting items before completing the checklist and 60% (SD 23%) after the checklist.

Eight manuscripts in the before group (40%, *n*=20) had titles that met guideline requirements, 6 (30%) that partially met requirements, and 6 (30%) that contained no information stipulated in the guideline item for title. Most guidelines stipulate describing the study design in the title as the main or only requirement. Two manuscripts in the “after” group improved their titles by adding information.

## Discussion

### Individual user feedback

The results from the survey suggested that users found the GoodReports website usable and useful. Most respondents (74%) reported making edits to their manuscript after using it and could back this up with examples of what they changed. The common objections and comments suggest users would find checklists more useful if they were shorter, easier to understand, and more applicable. Users also suggested that GoodReports be implemented at an earlier stage of writing.

### Questionnaire performance

Our questionnaire gave appropriate advice to 73% of users, so there is room for improvement. Sometimes the questionnaire had no recommendation where we thought there was an appropriate checklist available. Finding the right reporting guideline is not a straightforward task for either authors or the journals that endorse or even require authors to submit them. For example, a study of the effectiveness of a web-based tool to improve the reporting of randomised trials revealed that editorial staff were often unable to correctly identify a randomised trial based on what was reported in submitted manuscripts [11].

A common issue was authors responding that they were collecting exclusively qualitative data when they were either collecting exclusively quantitative data or both qualitative and quantitative data. As the question is quite long, people may have missed or misunderstand the word “exclusively.” We will think carefully about how to direct researchers who may collect both quantitative and qualitative data in one study.

Another common issue was authors of protocols either receiving no recommendation or an inappropriate recommendation. There are few reporting guidelines available for writing protocols for different study designs, and those that do exist often have restrictive usage licences. In the short term, we will adjust the questionnaire to recommend that authors of protocols use the appropriate reporting guideline for a completed study of the same design.

As we plan to add more reporting guidelines to the GoodReports database, future versions of the questionnaire could offer authors lists of options to help identify guidelines based on the design (e.g., type of trial or observational study), type of intervention or exposure (e.g., nutrition or psychological intervention), type of outcomes measured (e.g., economic or health equity), and focus of desired healthcare improvement (e.g., health policy or service delivery). Common study designs that cannot be matched to a reporting guideline could help direct future guideline development by indicating the largest need.

### Rates of submission

Partnering with *BMJ Open* and Penelope.ai was an effective way to attract users to www.goodreports.org and observe author behaviour. Authors who chose to use Penelope.ai for a paper check were more likely to submit a recommended reporting checklist with their submission, giving the editorial team one less thing to chase. A before-and-after study across four speciality medical research journals testing the previous version of the GoodReports questionnaire [29] also found that its use during submission was associated with improved author identification of the relevant reporting guidelines for their study type [32]. We cannot comment on causality here, as the data are all observational.

We found a discrepancy between how many manuscripts were identified as missing checklist submissions by the *BMJ Open* staff (10/69) and our team (16/69), when focusing on manuscripts that had been checked by Penelope.ai. We were unable to check the *BMJ Open* staff decisions for the rest of the manuscripts submitted in the 6-day window. The *BMJ Open* staff were unaware of whether an author had used Penelope.ai. It is therefore likely that they misclassified checklist needs for a similar proportion of manuscripts that had not been submitted to Penelope.ai. This is useful data for journals that plan to ask their staff to enforce reporting guideline policies. Journal staff may benefit from tailored training in identifying a manuscript’s study design and an appropriate reporting guideline. This also reminds us that it is insufficient to rely on one group within the research community to enforce reporting standards.

### Completeness of reporting

A quarter of authors made changes to their title or methods section after completing a GoodReports reporting checklist. Examples of significant changes included the addition of a paragraph on data management and a paragraph on power calculation. However, any change at all was rare, and the maximum changes recorded was six in one of the twenty manuscripts. On average, 40% of items were still missing from the methods section after completing an appropriate reporting checklist. The three systematic review protocols were the best reported manuscripts in our sample, but still missed several items after completing the PRISMA-P checklist. One quality improvement study had completed the SQUIRE checklist, but our assessors were unable to find any of the required items in the methods section. It is possible that the items had been reported in another section of the manuscript, which the assessors did not see. These results contrast with the user feedback study, where three-quarters of respondents said they had made changes after using a reporting checklist.

In this small sample, submission of a reporting checklist with an article did not indicate the article had been completely reported. The higher rate of checklist submission by authors who received a reporting guideline recommendation did not correspond with a higher rate of more complete manuscripts. It is plausible that recommending that authors use a reporting guideline at the point of submission is too late. By this stage, authors may not have the time, ability, or motivation to make substantial edits and clear changes with their co-authors. This problem will particularly apply to the methods section of completed studies. If key elements of the methods specified in the reporting checklist have not been carried out or recorded, they cannot be reported.

Much of the focus for promoting reporting guidelines has been via journals, specifically via the submission process and peer review [12]. However, reporting guidelines are designed to be used to direct early writing, not just as checklists after writing is finished. A writing tool based on CONSORT, called COBWEB, significantly increased the completeness of reporting of trial information [33]. Authors may therefore find GoodReports more useful during writing, rather than at article submission stage.

Potential ways to reach authors earlier in the writing process include contacting authors that publish Registered Reports [34], preprints [35], and protocols and working with researcher support organisations such as Author Aid [36].

Authors may also be more likely to react positively to reporting guidance even earlier in the research process, at funding application, protocol stage, or before data collection, rather than when writing up results. Seeing the essential information needed in a good study report early in the research process means researchers can adjust their plans if needed.

## Limitations

The data collected for each study was observational using convenience samples determined by journal editorial systems and our team’s availability to assess manuscripts. The data collected and described in the user survey was limited by the relatively low response rate you would expect from an optional user survey conducted amongst busy research professionals. Data were used to describe feasibility and user experience and to inform future development and experimental research. We cannot state whether GoodReports is an effective intervention until we test it in a randomised trial [37].

The author feedback data, including ideas on how to improve the tool, was collected from authors who chose to use Penelope.ai and try out the GoodReports tool. These people would be broadly representative of *BMJ Open* and Penelope.ai’s other journal clients, but not representative of all authors. However, the respondents were likely to be more representative than previous author experience surveys, which have generally been small, focused on a single reporting guideline, and included mainly European and US respondents [38–41]. Eighteen percent of the Penelope.ai users who responded to the question about how to improve GoodReports mentioned changes they would want to see in Penelope.ai. At least this proportion of survey respondents may have conflated the two tools and were commenting on Penelope.ai rather than GoodReports throughout the survey. However, as Penelope does not make suggestions relating to reporting completeness, so we can be sure that manuscript changes seen in the before-and-after study were the result of GoodReports.

For pragmatic reasons, we restricted our assessment of reporting completeness to the methods section and title, which may have skewed the effect of exposure to GoodReports. However, the primary purpose of the manuscript completeness assessment study was to pilot the assessment task with the team. This will inform the assessment protocol for a future randomised trial where we will assess both the methods and results sections [37].

The assessors could not be blinded because the “before” file format was different from the “after” file format. Any potential for bias was mitigated by the objective outcome measure of whether the two versions of text were identical or whether text had been added.

We were heartened by one user’s comment: “I will refer to all checklists to write my report clearly and fully next time”. We are optimistic that the tools we are developing will help authors, increase the adoption of reporting guidelines, and, ultimately, reduce research waste.

## Conclusion

This paper described steps in developing a tool to help authors access appropriate reporting guidance more easily. The data gathered suggested that authors using the tool at submission were more likely to submit a completed reporting guideline checklist and that the tool successfully suggested the correct checklist. However, very few authors used the checklists to add information to their papers. These results underline the need for reporting guidance to be introduced early in the writing process. To better support authors beginning to write, we will investigate delivering reporting guidance in article templates.

## Supplementary Information

**Additional file 1 : Table S1.** Themes and representative free-text responses (in italics) to the question “How could we make www.goodreports.org more useful?”. **Table S2.** Themes and representative comments (in italic) about changes made or reasons for not making changes. **Table S3.** Summary of possible reasons for inappropriate recommendations, with potential solutions

## Data Availability

The datasets generated and/or analysed during the study are available on the Open Science Framework GoodReports development site: https://osf.io/2pejk/ 1. Individual user feedback: https://osf.io/h35aw/ 2. Performance of the questionnaire in helping authors find the most appropriate reporting guideline for their work: https://osf.io/fmq9s/ 3. Exposure to GoodReports.org and rates of submission of a completed reporting checklist: https://osf.io/6ajh3/ 4. Exposure to GoodReports.org and completeness of reporting (before-and-after study): https://osf.io/v435t/ Protocol for assessors: https://osf.io/t8jpm/ Sample data collection form: https://osf.io/jnk8h/

## Abbreviations

EQUATOR: Enhancing Quality and Transparency of Health Research
PRISMA-P: Preferred Reporting Items for Systematic reviews and Meta-Analyses for Protocols
SRQR: Standards for Reporting Qualitative Research
SPIRIT: Standard Protocol Items: Recommendations for Interventional Trials
CONSORT: Consolidated Standards of Reporting Trials
STROBE: STrengthening the Reporting of OBservational studies in Epidemiology
TRIPOD: Transparent Reporting of a multivariable prediction model for Individual Prognosis Or Diagnosis
STARD: Standards for Reporting Diagnostic Accuracy
MOOSE: Meta-analyses Of Observational Studies in Epidemiology
