# Supplementary material for: GoodReports: developing a website to help health researchers find and use reporting guidelines
Source: BMC Med Res Methodol. 2021 Oct 17;21:217. doi: 10.1186/s12874-021-01402-x (PMC8520646; doi:10.1186/s12874-021-01402-x)
Supplement: Supplementary file 1 — Additional file 1 : Table S1. Themes and representative free-text responses (in italics) to the question “How could we make www.goodreports.org more useful?”. Table S2. Themes and representative comments (in italic) about changes made or reasons for not making changes. Table S3. Summary of possible reasons for inappropriate recommendations, with potential solutions [file 12874_2021_1402_MOESM1_ESM.docx]

**GoodReports: developing a website to help health researchers find and use reporting guidelines**

# Supplementary tables

#### Table S1: Themes and representative free-text responses (in italics) to the question "How could we make [www.goodreports.org](http://www.goodreports.org) more useful?"

| **Themes** | | Number of responses |
| --- | --- | --- |
| Simplification (e.g., shorten the checklists and their items and clarify the item’s wording) | | 26/93 (28%) |
|  | - *By simplifying it and much shortening* - *A bit shorter would be good. Too many questions on data monitoring and storage* - *The resulting interface is not clear enough* |  |
| Applicability (e.g., include a wider variety of guidelines and remove non-applicable reporting items for that study) | | 17/93 (18%) |
|  | - *can include more checklists that are commonly used.* - *provide more specific checklist items and give example if it is possible, to avoid us to get confused maybe by providing feedback* - *More options for a specific report. Our report was an animal based cadaveric study looking at accuracy of drill guides. We were unsure which category it should fall under.* - *There should be an explanation that not all questions might be applicable.* |  |
| Give more information (e.g., examples of good reporting, definitions of reporting items, and more instruction on how to use the checklist) | | 15/93 (16%) |
|  | - *Give more detailed information about filling out checklist items. Some items are not very clear.* - *Definitions of all of the terms as hyperlinks.* - *Add boxes on the checklist to add the corresponding page number* - *have a key to explain basic terms such as sensitivity analysis for first time users, forest plot* - *Online video tutorials or workshops would help a lot* |  |
| Technical improvements to the GoodReports website | | 11/93 (12%) |
|  | - *a window that allows you to view your manuscript in real time while going through the checklist will be highly valuable.* - *Put in the checklist automatically* - *Mark in the text what corresponds to each item in the list* |  |
| Promotion (e.g., suggestions to reach more authors and at earlier stage of writing) | | 10/93 (11%) |
|  | - *Easily accessible... Inform through social media platforms*   *make available for all journal submissions*   - *You could make more visible to the junior researchers.* - *Dissemination in universities and research centers* - *Encourage people to use the criteria early in the writing process (I have, which probably is why I only changed one thing)* |  |
| Suggestions for specific guidelines | | 2/93 (2%) |
|  | *For a systematic review, the PRISMA guidelines can also be used rather than the MOOSE.* |  |
| Unclear (we were not certain what the user meant) | | 12/93 (13%) |
|  | - *by making/and more highlighting the specific parts for readers.* - *integrating them in reporting guidelines* - *Reducing the burden of comparison a manuscript with others* |  |

#### Table S2: Themes and representative comments (in italic) about changes made or reasons for not making changes.

| **Themes** | Number of responses |
| --- | --- |
| **Respondents that *had* made changes** | **198/267 (74%)** |
| Provided reasonable detail about what they had changed | 51/198 (26%) |
| - *Some precision on the definition of the dimension of of intervention we were studying* - *extra detail added regarding blinding/allocation of intervention* - *I added the consent form, protocol contributor information, role of study sponsor, adherence efforts, criteria for discontinuation, and updated safety measures and data security descriptions.* - *Added more detail about the conceptual framework used* - *Add data management and confidentiality and harms to the manuscript* - *included how the sample size was determined* - *mainly just adding more detail e.g. about method of randomisation* |  |
| Provided non-specific information about what they had changed | 115/198 (58%) |
| - *The structure of manuscript and details in each section* - *Some of the items that are stated in the STROBE but were not written in the original manuscript* - *Made sure that each sub-title conformed to the checklist suggested nomenclature* - *A lot! Added info and checked other components* - *More details that were missing in some sections* |  |
| Did not provide details about what they had changed | 32/198 (16%) |
| **Respondents that *had not* made changes** | **69/267 (26%)** |
| Had already followed a reporting guideline when writing | 16/69 (23%) |
| No edits required because they had already reported all items (but did not explicitly mention following a reporting guideline) | 31/69 (45%) |
| Did not want to | 7/66 (10%) |
| - *I did not have the time finish it so I changed a journal* - *too much TROUBLE* - *I don’t accept the changes* |  |
| Unclear (e.g., "*It's OK*") | 7/66 (10%) |
| No answer | 8/66 (12%) |

#### Table S3: Summary of possible reasons for inappropriate recommendations, with potential solutions

| **Reporting guideline inappropriately recommended** | **# of users** | **Possible reason for mismatch** | **Potential solution** |
| --- | --- | --- | --- |
| SRQR for reporting qualitative research | 7 | All responded that their study used exclusively qualitative data. However, although most of the studies did collect qualitative data, they also collected quantitative data and used a standard design that would have better fit another reporting guideline (e.g., CONSORT or STROBE). As the current question in the finder is very long, authors may have missed the word “exclusively.” | Split the question into two to accommodate studies with both qualitative and quantitative data. Allow the finder to recommend a combination of reporting guidelines. |
| SPIRIT for reporting protocols of randomised trials | 3 | All responded that they were reporting a protocol of a clinical trial, but their protocol was for a different study design. There are only reporting guidelines for protocols for two study designs (clinical trials and systematic reviews). Authors writing other kinds of protocol may have decided to choose one of the presented designs rather than selecting “other” on the final question. | Give more useful advice to authors writing protocols for which no guideline currently exists or is openly available. It may be more useful to direct authors to the design’s reporting guideline instead, with an instruction to focus on the methods. |
| STARD for reporting diagnostic test accuracy studies | 3 | All responded that they were investigating a diagnostic test.  Two were studies that focused on tests and screening but were not primary studies of diagnostic test accuracy (DTA). They would have been better matched by STROBE.  The third was a meta-analysis of DTA studies. They were not matched with PRISMA as they had not used a systematic review design. | Make the question directing people to STARD more precise, to be clear that it does not refer to all studies focused on tests and screening.  Include a question to identify studies that use meta-analysis without systematic review. |
| STROBE cohort | 2 | Both studies were correctly identified as using observational study designs, with mismatch in one case on which of the STROBE sub-designs applied.  One study was a cross-sectional study of a previous research cohort.  The second was a retrospective cohort study on the results of a screening test which we incorrectly judged should have been matched with STARD. | Include clearer explanations, potentially with graphics, to help authors identify which type of observational study they have done. |
| CONSORT for reporting randomised trials | 2 | The authors of the first study indicated that they used an experimental design. However, the study was a survey with no investigated intervention.  The authors of the second study correctly identified their work as experimental. However, they responded that two groups were being compared when they used a single-arm study design. | Include a clearer explanation of the difference between experimental and observational research.  Direct authors of phase 1 trials better. |
| CHEERS for economic evaluations | 1 | Although this was a cohort study the study participants were selected on basis of resource use. The authors therefore indicated that the study was looking at economic aspects of healthcare. | Clarify that the question regarding economic aspects only refers to primary outcomes. |
| MOOSE for meta-analysis of observational studies | 1 | Although the author’s responses were reasonable, the study included meta-analyses of both trials and observational research. PRISMA would have been a better fit. | Include advice on what to do if your manuscript reports multiple study types. |
| PRISMA-P for protocols of systematic reviews | 1 | Although the authors responded that they were writing a protocol for a systematic review, they were planning a “realist” review. There is no appropriate reporting guideline for this design. | Give more useful advice to authors writing protocols for which no guideline currently exists or is openly available. |
| STROBE case-control | 1 | The study was correctly identified as observational research, with mismatch on which of the 3 STROBE sub-designs applied. This before-and-after study used a cohort design, not a case-control design as selected. As the study related to a new care management system, SQUIRE might also have been helpful. | Include clearer explanations, potentially with graphics, to help authors identify which type of observational study they have done.  Include advice on what to do if more than one reporting guideline would apply to your manuscript. |
| **Recommendation that there is no appropriate checklist** | **# of users** |  |  |
| **Our recommendation** |  |  |  |
| SPIRIT | 2 | 1 of the manuscripts was a pilot randomised trial, but the author response indicated that their article was a protocol.  The authors of the other manuscript identified it as a protocol, but then selected “other” instead of clinical trial. The trial was community-based not clinic-based, so their response was technically correct | Expand explanation of what a protocol is.  Change “clinical trial” to “randomised trial” in the questionnaire |
| CONSORT | 1 | The response did not logically match the study design. The authors responded to the initial screening question that they were not doing health or biomedical research, although the article reported secondary analyses of a randomised clinical trial |  |
| STROBE Cohort | 1 | The authors chose "other" from options of article type (rather than case report, protocol, systematic review, or research article). However, the work presented initial findings of a birth cohort | Clarify what ‘research’ means, perhaps by adding examples (e.g. experimental or observational).  Add more helpful feedback than "no guideline" for these responses. |
| STROBE Cross-sectional | 1 | The authors did not give any further response after saying that the study collected data from people.  They may have struggled with the next question, "What are you investigating?" As they conducted observational/epidemiological research, the correct option was “factors affecting health.” This option may be too vague. | Clarify what ‘factors affecting health’ refers to, to make the connection to epidemiological/observational research clearer. |
| STROBE Case-control | 1 | The authors correctly responded they were writing a protocol and provided no further responses. Their paper was a protocol for a case-control study, so STROBE case-control would have been a good fit | Give more useful advice to authors writing protocols for which no guideline currently exists or is openly available |
